# Supplementary material for: From sequence to enzyme mechanism using multi-label machine learning
Source: BMC Bioinformatics. 2014 May 19;15:150. doi: 10.1186/1471-2105-15-150 (PMC4229970; doi:10.1186/1471-2105-15-150)
Supplement: Additional file 2 — Java code of ml2db. Additional file ml2db_code.tar.gz contains the Java source code to run the multi-label machine learning experiments and save the results to database. The code’s Javadoc is included. [file 1471-2105-15-150-S2.zip › additional file 2/ml2db/ml2db/doc/test/ml2db/weka/InstancesFillerTest.html]

InstancesFillerTest


JavaScript is disabled on your browser.


- Overview
- Package
- Class
- Use
- Tree
- Deprecated
- Index
- Help

- Prev Class
- Next Class

- Frames
- No Frames

- All Classes

- Summary:
- Nested |
- Field |
- Constr |
- Method

- Detail:
- Field |
- Constr |
- Method


test.ml2db.weka

## Class InstancesFillerTest

- java.lang.Object
- - junit.framework.Assert
  - - junit.framework.TestCase
    - - test.ml2db.weka.InstancesFillerTest

- All Implemented Interfaces:
  :   junit.framework.Test

  ---

    

  ```
  public class InstancesFillerTest
  extends junit.framework.TestCase
  ```

  Class

  Version:
  :   22 Feb 2008

  Author:
  :   Luna De Ferrari ldf(at)st-andrews.ac.uk

- - ### Constructor Summary

    Constructors

    Constructor and Description

    `InstancesFillerTest()`
  - ### Method Summary

    Methods

    | Modifier and Type | Method and Description |
    | --- | --- |
    | `static InstancesFiller` | `instancesFactory()` |
    | `void` | `testWriteInstance()` public void testFillNonExistingInstances() { InstancesFactory factory = InstancesFactoryTest.instancesFactory(); SparseInstance instance = new SparseInstance(factory.getDataSet() .getAttributes().size()); // set the dataset (Instances) for the instance instance.setDataset(factory.getDataSet().getInstances()); // fill instance attribute values instance = factory.fillInstance("empty", instance); assertEquals("@attribute att1 {n,y}", instance.attribute(0).toString()); assertEquals("@attribute att3 {n,y}", instance.attribute(2).toString()); assertEquals(4, instance.classIndex()); assertEquals("NaN", Double.toString(instance.classValue())); assertEquals(4, instance.index(0)); assertEquals(5, instance.numAttributes()); assertEquals(1, instance.numValues()); assertEquals("{4 ?}", instance.toString()); } |

    - ### Methods inherited from class junit.framework.TestCase

      `countTestCases, getName, run, run, runBare, setName, toString`
    - ### Methods inherited from class junit.framework.Assert

      `assertEquals, assertEquals, assertEquals, assertEquals, assertEquals, assertEquals, assertEquals, assertEquals, assertEquals, assertEquals, assertEquals, assertEquals, assertEquals, assertEquals, assertEquals, assertEquals, assertEquals, assertEquals, assertEquals, assertEquals, assertFalse, assertFalse, assertNotNull, assertNotNull, assertNotSame, assertNotSame, assertNull, assertNull, assertSame, assertSame, assertTrue, assertTrue, fail, fail, failNotEquals, failNotSame, failSame, format`
    - ### Methods inherited from class java.lang.Object

      `equals, getClass, hashCode, notify, notifyAll, wait, wait, wait`

- - ### Constructor Detail


    - #### InstancesFillerTest

      ```
      public InstancesFillerTest()
      ```
  - ### Method Detail


    - #### testWriteInstance

      ```
      public void testWriteInstance()
      ```

      public void testFillNonExistingInstances() { InstancesFactory factory =
      InstancesFactoryTest.instancesFactory(); SparseInstance instance = new
      SparseInstance(factory.getDataSet() .getAttributes().size());
      // set the dataset (Instances) for the instance
      instance.setDataset(factory.getDataSet().getInstances());
      // fill instance attribute values instance =
      factory.fillInstance("empty", instance);
      assertEquals("@attribute att1 {n,y}", instance.attribute(0).toString());
      assertEquals("@attribute att3 {n,y}", instance.attribute(2).toString());
      assertEquals(4, instance.classIndex()); assertEquals("NaN",
      Double.toString(instance.classValue())); assertEquals(4,
      instance.index(0)); assertEquals(5, instance.numAttributes());
      assertEquals(1, instance.numValues()); assertEquals("{4 ?}",
      instance.toString());
      }


    - #### instancesFactory

      ```
      public static InstancesFiller instancesFactory()
      ```


- Overview
- Package
- Class
- Use
- Tree
- Deprecated
- Index
- Help

- Prev Class
- Next Class

- Frames
- No Frames

- All Classes

- Summary:
- Nested |
- Field |
- Constr |
- Method

- Detail:
- Field |
- Constr |
- Method
